# Serum IL-6, IL-1b, IL-8, and cerebrospinal fluid biochemical profiles in patients with lateral skull base temporal bone fractures and cerebrospinal fluid leak

**DOI:** 10.5937/jomb0-56530

**Published:** 2025-10-28

**Authors:** Jianhua Zhu, Juhua Qian

**Affiliations:** 1 Affiliated Aoyang Hospital of Jiangsu University, Department of Head and Neck Surgery, Aoyang Hospital, Zhangjiagang, Suzhou, Jiangsu 215600, China

**Keywords:** IL-6, IL-1b, IL-8, nutritional care, lateral skull base temporal bone fractures, cerebrospinal leakage, cranial reconstructive therapy, lumbar drainage, clinical outcomes, IL-6, IL-1b, IL-8, nutritivna nega, prelomi temporalne kosti baze lobanje, curenje cerebrospinalne tečnosti, terapija rekonstrukcije lobanje, lumbalna drenaža, klinički ishodi

## Abstract

**Background:**

This study evaluates the impact of cranial reconstruction therapy combined with lumbar drainage on inflammatory cytokine levels (IL-6, IL-1b, IL-8), serum albumin, and cerebrospinal fluid (CSF) biochemical markers in patients with lateral skull base temporal bone fractures and cerebrospinal fluid leakage. It also assesses the role of nutritional and psychological care in patient recovery.

**Methods:**

A total of 130 patients with temporal bone fractures and CSF leakage who underwent craniotomy repair surgery between May 2022 and May 2024 were enrolled. Patients were randomly assigned to either a control group (CG), receiving craniotomy and standard nutritional care, or an observation group (OG), receiving cranial reconstruction combined with lumbar drainage and nutritional intervention. CSF protein, glucose, and chloride levels were measured on postoperative day 7. Systemic inflammation was assessed by measuring temperature, WBC count, CRP, IL-6, IL-1b, and IL-8 at 7 and 15 days postoperatively. Nutritional status was evaluated using serum albumin (ALB), total protein (TP) levels, and Subjective Global Assessment (SGA) scores before and after treatment.

**Results:**

By postoperative day 7, IL-6 levels were significantly lower in OG (10.5±2.3 pg/mL) compared to CG (18.2±4.1 pg/mL), IL-1b was 8.4±1.7 pg/mL in OG versus 14.3±3.5 pg/mL in CG, and IL-8 levels were 15.2±3.1 pg/mL in OG versus 22.5±4.2 pg/mL in CG (all P&lt;0.05). CSF protein levels were lower in OG, and glucose and chloride levels were higher (P&lt;0.05), with all values within the normal range. Inflammatory markers (IL-6, IL-1b, IL-8, WBC, and CRP) showed a further reduction by day 15 (P&lt;0.01) in OG. Serum albumin levels were significantly higher in OG postoperatively (P&lt;0.01). No significant differences were observed between groups for TP and SGA scores.

**Conclusions:**

Cranial reconstruction therapy combined with lumbar drainage accelerates the resolution of inflammation (IL-6, IL-1b, IL-8), improves cerebrospinal fluid biochemical markers, and enhances nutritional recovery (albumin levels), leading to better clinical outcomes. Including nutritional and psychological support further enhances patient recovery and quality of life. These findings underscore the importance of monitoring inflammatory and nutritional biomarkers to optimise postoperative management in patients with temporal bone fractures and CSF leakage.

## Abstract (sr)

**Uvod:**

Ova studija procenjuje uticaj terapije rekonstrukcije lobanje u kombinaciji sa lumbalnom drenažom na nivoe inflamatornih citokina (IL-6, IL-1b, IL-8), serumskog albumina i biohemijske parametre cerebrospinalne tečnosti (CST) kod pacijenata sa prelomima temporalne kosti baze lobanje sa lateralne strane i curenjem cerebrospinalne tečnosti. Takođe, procenjuje se uloga nutritivne i psihološke nege u oporavku pacijenata.

**Metode:**

U studiju je uključeno ukupno 130 pacijenata sa prelomima temporalne kosti i curenjem CST , koji su bili podvrgnuti hirurškoj rekonstrukciji lobanje u periodu od maja 2022. do maja 2024. godine. Pacijenti su nasumično podeljeni u kontrolnu grupu (CG), koja je primala standardnu hiruršku rekonstrukciju i nutritivnu negu, i posmatranu grupu (OG), koja je imala rekonstrukciju lobanje kombinovanu sa lumbalnom drenažom i nutritivnom intervencijom. Nivoi proteina, glukoze i hlorida u CST mereni su sedmog dana nakon operacije. Sistemsku inflamaciju procenjivali smo merenjem telesne temperature, broja leukocita, CRP-a, IL-6, IL-1b i IL-8 sedmog i petnaestog dana nakon operacije. Nutritivni status procenjivan je pomoću nivoa serumskog albumina (ALB), ukupnih proteina (TP) i skora Subjektivne globalne procene (SGA) pre i nakon tretmana.

**Rezultati:**

Sedmog postoperativnog dana, nivo IL-6 bio je značajno niži u OG (10,5±2,3 pg/mL) u poređenju sa CG (18,2±4,1 pg/mL), nivo IL-1b iznosio je 8,4±1,7 pg/mL u OG naspram 14,3±3,5 pg/mL u CG, dok je nivo IL-8 bio 15,2±3,1 pg/mL u OG u odnosu na 22,5±4,2 pg/mL u CG (svi P&lt;0,05). Nivoi proteina u CST bili su niži u OG, dok su nivoi glukoze i hlorida bili viši (P&lt;0,05), pri čemu su sve vrednosti bile unutar referentnih granica. Infla matorni markeri (IL-6, IL-1b, IL-8, leukociti i CRP) pokazali su dodatno smanjenje do petnaestog dana (P&lt;0,01) u OG. Nivoi serumskog albumina bili su značajno viši u OG nakon operacije (P&lt;0,01). Nije bilo značajnih razlika između grupa u pogledu ukupnih proteina (TP) i SGA skorova.

**Zaključak:**

Terapija rekonstrukcije lobanje u kombinaciji sa lumbalnom drenažom ubrzava smanjenje inflamacije (IL-6, IL-1b, IL-8), poboljšava biohemijske parametre cerebrospinalne tečnosti i unapređuje nutritivni oporavak (nivo albu mina), što dovodi do boljih kliničkih ishoda. Uklju či va nje nutritivne i psihološke podrške dodatno poboljšava opora vak pacijenata i kvalitet života. Ovi nalazi naglašavaju zna čaj praćenja inflamatornih i nutritivnih biomarkera radi optimizacije postoperativnog lečenja kod pacijenata sa prelomima temporalne kosti i curenjem cerebrospinalne tečnosti.

## Introduction

Most patients with craniocerebral trauma suffer from skull base fractures. The lateral skull base region is structurally complex, bearing the cranium above and the neck below, and is rich in blood vessels, nerves, and organs of locus coeruleus. The temporal bone is a crucial structure in lateral skull base fractures. It is the thickest structure in the skull base, so the risk of vascular and neurological injury is more significant in temporal bone fractures (TBF) than in other skull fractures [1]. Data proves that skull fractures account for 4% of head trauma, while temporal bone fractures account for 14–22% of skull fractures [2] [3]. The main causes of temporal bone fractures are traffic accidents, falling objects and falls, with traffic accidents accounting for the largest proportion, at 31% [4]. In addition, TBF is more common in men (~80%), and the type of fracture is mainly unilateral, with only a 12% chance of being bilateral [5] [6]. Neurological symptoms after TBF include hearing loss, vertigo, facial paralysis, and cerebrospinal fluid leakage [7]. Of these, cerebrospinal leakage is up to 45% more likely to occur in patients with temporal bone fractures, and ear leakage is one of the most common manifestations of temporal bone fractures [8]. Patients with cerebrospinal leaks are highly susceptible to intracranial infections leading to bacterial meningitis, with a prevalence of 88 per cent [9]. Multiple studies show antibiotics reduce meningitis rates [10] [11]. Accordingly, antibiotics are widely used in patients with skull base fractures combined with cerebrospinal leakage.

Lateral skull base temporal bone fractures, often accompanied by cerebrospinal fluid (CSF) leakage, present a significant clinical challenge due to the potential for severe complications, including infection, meningitis, and delayed recovery. Inflammatory responses following such injuries are complex and multifactorial, with cytokines such as IL-6, IL-1β, and IL-8 playing critical roles in mediating both the acute-phase response and the immune system’s defence mechanisms [12]. IL-6, a pro-inflammatory cytokine, is known to be elevated in response to trauma and surgical interventions, contributing to systemic inflammation and tissue repair [13]. Similarly, IL-1β, another key pro-inflammatory mediator, drives the local immune response and induces fever, pain, and further inflammatory cascades, while IL-8 facilitates the recruitment of neutrophils to sites of injury or infection [14]. Given their involvement in these processes, these interleukins are valuable biomarkers for assessing the severity of inflammation and potential complications such as infection and prolonged recovery in patients with skull base fractures.

This combination is hypothesised to be more effective due to its potential to reduce intracranial pressure (ICP) and improve cerebrospinal fluid (CSF) dynamics. It could subsequently decrease the inflammatory response and promote faster recovery. By alleviating ICP and facilitating the flow of CSF, lumbar drainage may help resolve inflammation more rapidly and promote quicker healing, offering a dual approach to the complex challenges associated with temporal bone fractures and CSF leakage.

In addition to cytokines, serum markers such as albumin provide valuable insights into the patient’s overall recovery. Albumin levels reflect not only the patient’s nutritional status but also the extent of systemic inflammation, as hypoalbuminemia is often linked to poorer outcomes in post-traumatic or post-surgical settings [15] [16]. The measurement of IL-6, IL-1β, IL-8, and albumin in the context of lateral skull base fractures and CSF leakage offers an integrated approach to understanding both the local and systemic inflammatory responses [17]. These biomarkers may be early indicators of complications such as infection, delayed wound healing, and neurological damage. As such, they could be pivotal in refining therapeutic strategies and improving patient prognosis by enabling more precise monitoring of inflammation and recovery during the critical postoperative period. This article explores the relevance of these markers in assessing and managing patients with temporal bone fractures, highlighting their potential to predict and mitigate adverse clinical outcomes.

Temporal bone fracture with cerebrospinal fluid leakage is mostly self-healing with conservative treatment, and cranial repair surgery is required when the leakage persists for more than 2–3 weeks [18]. The main entry points for surgical repair of temporal bone cerebrospinal fluid leaks include the middle cranial fossa, mastoid and combined approaches. The advantages of the transmastoid approach are preservation of hearing and good identification and repair of the fistula [19]. There is still some chance of repair failure or persistent fluid leakage. Continuous drainage with lumbar pool placement is a widely used cerebrospinal fluid drainage method that promotes the healing of intracranial fistulae by reducing intracranial pressure.

In this study, we combined nutritional care with surgical reconstructive treatment to compare clinical outcomes, complications and QOL between different surgical treatments.

## Materials and methods

### Study subjects

One hundred and thirty patients with lateral skull base temporal bone fracture with cerebrospinal leakage who underwent intracranial repair surgery from May 2022 to May 2024 in our hospital were selected as study subjects. They were randomly divided into CG and OG, 65 in each group. The inclusion and exclusion criteria for patient selection were as follows. Inclusion Criteria: (1) Diagnosed as the case of lateral skull base temporal bone fracture with cerebrospinal rhinorrhea by cranial CT and MRI; (2) Conscious and cooperating with treatment. Exclusion criteria: (1) combination of malignant tumour and autoimmune disease; (2) abnormal cardiac and hepatic functions; (3) coagulation disorders; (4) abnormal blood sugar. The hospital’s Ethics Committee approved this study.

### Treatment protocol

Both groups were given surgical treatment plus nutritional care. CG was treated with craniotomy, and OG was treated with craniotomy repair and reconstruction combined with lumbar pool placement for continuous drainage, and antibiotics were given in the perioperative period.

Craniotomy reconstruction treatment: Both groups of patients were treated with craniotomy repair and reconstruction, and patients with cerebrospinal fluid leakage from temporal bone fracture of the lateral skull base were repaired through the mastoid approach. Preoperative bed rest, head elevation of 30 degrees, fasting 6 h after the administration of surgical treatment. Tracheal intubation and general intravenous combined anaesthesia were given. An incision was made behind the ear, and the temporal muscle fascia was left in reserve. The mastoid was exposed, and a finish-wall mastoidotomy was performed to open the tympanic sinus, remove part of the bone of the tunica and expose the dura mater. The dura mater was pushed upwards to reveal the fistula. In patients with dural defects >1 cm, cartilage support is re quired, the mastoid cavity is filled with fat, and the surface is covered with temporalis muscle fascia. Once the repair is complete, the incision behind the ear is closed in layers with a pressure dressing. The middle cranial fossa approach was used for patients with multiple basket openings or dural defects >2 cm, with the bone window as close to the bottom of the fossa as possible. The fistula was filled with fat to cover the temporal myofascial membrane, and then the wound was closed tightly with layered sutures. Postoperatively, patients were strictly required to maintain bed rest for 2 weeks to promote healing. In addition, intracranial pressure (ICP) reduction measures were implemented, and perioperative antibiotic therapy was administered to prevent infection. The antibiotics used included ceftriaxone (2 g IV) and metronidazole (500 mg IV), which were given for 7 days to help control the risk of infection following the surgery.

Lumbar pool tube placement for continuous drainage: The OG was prevented from using the large lumbar pool drainage tube after surgery. The puncture bursts into the subarachnoid space, and the silicone drainage tube is sent into the large pool of the lumbar pool. After fixing the drainage catheter, a tee tube and drainage bag are connected to the end of the drainage tube. This method reduces intracranial pressure by human intervention and allows the fistula to heal independently.

Nursing care: 1) Nutritional care: postoperative patients should consume high protein, high calorie and easy-to-digest food and supplement sufficient vitamins and fruits daily to keep the intestinal tract unobstructed. If there is no bowel movement, a corkscrew or laxative can be given to unblock the intestinal tract so as not to force defecation to increase intracranial pressure. Early enteral nutrition can be given to patients who cannot eat by mouth. For comatose and frail patients, intravenous nutritional support can be given; for other patients, enteral nutrition can be carried out through the use of dental pads with transoral indwelling gastric tubes. 2) Psychological care: through health education, the professional knowledge of cerebrospinal fluid nasolacrimal leakage can be popularised to the patients and their families, and successful treatment cases can be introduced to alleviate the patient’s fears and anxieties and promote the recovery of their conditions.

### Observation indicators

**Clinical outcomes:** Statistics on cure rate, recurrence, and mortality of patients.

**Clinical symptoms:** The time to symptom relief, the time to infection control, and intracranial pressure at 7 d were counted.

**Clinical indicators of cerebrospinal fluid:** On postoperative day 7, levels of CSF protein, glucose, and chlorides were measured.

**Inflammation level:** To evaluate the inflammatory response, the patient’s temperature, white blood cell (WBC) count, C-reactive protein (CRP) levels, and levels of interleukins (IL-6, IL-1β, and IL-8) were tested at 7 and 15 days postoperatively. These interleukins are critical inflammatory markers that help predict postoperative complications, such as infection or delayed recovery. Elevated levels of IL-6, IL-1β, and IL-8 indicate heightened systemic inflammation and may correlate with longer recovery times or increased risk of complications.

Nutritional status: Patients were tested for pre- and postoperative serum albumin (ALB), total protein (TP) levels, and Subjective Global Assessment (SGA) levels. Serum albumin is a key indicator of nutritional status and is essential in inflammation regulation and tissue healing. Low albumin levels are often associated with malnutrition, which can adversely affect recovery in patients with temporal bone fractures. Monitoring ALB, in addition to TP and SGA, provides a comprehensive picture of the patient’s nutritional and inflammatory state.

**Complications:** Observe the probability of intracranial infection, fever, cerebrospinal fluid nasal leakage, intracranial hypertension, and other complications after treatment of patients.

**Negative emotions:** The SAS scale assesses the patient’s level of anxiety, and the SDS assesses the patient’s level of depression. The scores were inversely proportional to negative mood.

**Quality of life:** QOL was assessed using the SF-36 scale. The four dimensions of physical functioning, mental health, social functioning, and general health from the SF-36 scale were selected for assessment, and QOL was positively related to the score.

## Results

### Comparison of patients’ clinical data

Clinical data were analysed in terms of age, gender, cause of accident, severity of illness and clinical symptoms. The average age of CG patients was (45.1±11.9) years, of which 40 were male, and 25 were female; there were 46 cases of car accidents, 16 cases of falling objects, and 3 cases of injuries. The average age of OG patients was (46.5±10.68) years, of which 38 were male, and 27 were female; there were 49 cases of car accidents, 15 cases of falling objects, and 1 case of injuries. 49 cases, 15 cases of falling objects and 1 case of bruising. The injuries were classified into three grades according to the GCS score: heavy brain injury GCS 3–8, medium GCS 9–12, and light brain injury GCS 13–15. The rate of occurrence of cerebrospinal fluid leakage was higher in patients with temporal bone fractures. There was no significant difference in the general clinical data between the two groups of patients (P>0.05), as shown in Table 1.

**Table 1:** Demographic data of study participants.

Items		CG (n=65)	OG (n=65)	P-value
Age		45.1±11.9	46.5±10.68	0.482
Gender(male/female)		40/25	38/27	0.72
Causes of accidents	Traffic accidents	46	49	0.553
	Falling objects	16	15	0.837
	Bruise	3	1	0.619
Typology	Ear leakage	16	13	0.527
	Nose leakage	49	52	0.527
GCS score craniocerebral injury	light	18	21	0.566
	medium	36	36	1
	heavy	11	8	0.62

### Cranial reconstructive therapy combined with lumbar big-pool drainage improves clinical outcomes in patients

CG of the 65 patients, 57 patients were cured, 7 had recurrent cerebrospinal leakage of fluid, and one died due to intracranial infection. OG of the 65 patients, 63 patients were cured, 1 had recurrent cerebrospinal leakage of fluid, and there was no death. The cure rate of OG was significantly higher than that of OG (P<0.05, Table 2).

**Table 2:** Demographic data of study participants.

Groups	curable	failures	mortality
CG (n=65)	57 (87.69)	7 (46.15)	1 (1.54)
OG (n=65)	64 (96.92)	1 (1.54)	0 (0.00)
X^2^	4.298	4.795	-
p	0.038	0.029	-

### Cranial reconstructive therapy combined with lumbar big-pool drainage to relieve patients’ clinical symptoms

As shown in Table 3, the time to symptom relief, time to infection control, and intracranial pressure on the seventh postoperative day were lower in OG than in CG (P<0.05).

**Table 3:** Comparison of clinical symptoms.

Groups	Symptom<br>relief time<br>(d)	Infection<br>control time<br>(d)	Intracranial<br>pressure <br>(mmH_2_O)
CG (n=65)	5.48±2.13	12.83±5.41	139.74±19.89
OG (n=65)	4.11±2.02	7.16±4.04	128.03±19.67
t	3.763	6.77	3.375
P	<0.001	<0.001	<0.001

### Cranial reconstructive therapy combined with lumbar big-pool drainage for faster improvement of cerebrospinal fluid clinical indicators

**Figure 1:**
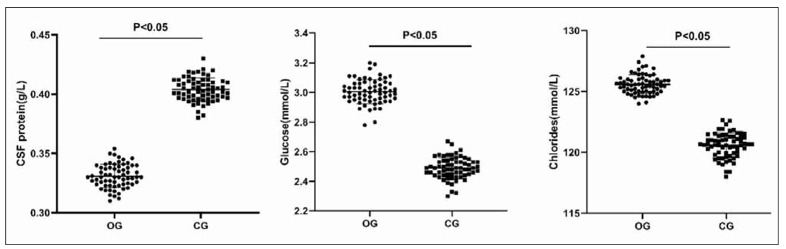
Evaluation of clinical indicators of cerebrospinal fluid.

As shown in Figure 1, the biochemical results on the seventh postoperative day showed that the level of CSF protein in OG was lower than that in CG, and the levels of glucose and chlorides were higher than those in CG (P<0.05). The biochemical index levels of OG were within the normal range (P<0.05).

### Cranial reconstructive therapy combined with lumbar big-pool drainage more rapidly improves inflammation levels in patients

**Figure 2:**
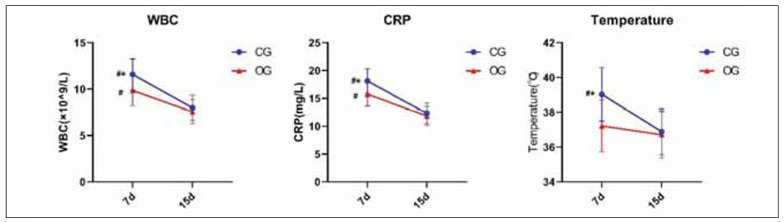
Evaluation of inflammation levels. ^#^P<0.05 represents 7 d versus 15 d. *P<0.05 represents CG versus OG.

As shown in Figure 2, the body temperature and WBC, CRP levels of OG patients were lower than those of CG at 7 d postoperatively (P<0.05), and there was no statistical significance between the two groups at 15 d postoperatively (P>0.05).

Additionally, IL-6, IL-1β, and IL-8 levels were significantly elevated in both groups at 7 d postoperatively, indicating an acute inflammatory response. However, OG patients exhibited a greater reduction in IL-6 and IL-1β levels by 15 d compared to CG (P<0.05), suggesting a more effective inflammatory resolution. IL-8 levels also showed a significant decline in OG compared to CG at 15 d (P<0.05), reinforcing the effectiveness of combined cranial reconstruction and lumbar drainage in controlling post-surgical inflammation (Table 4).

**Table 4:** Comparison of inflammatory markers.

Groups	IL-6<br>(pg/mL)<br>at 7 d	IL-6<br>(pg/mL)<br>at 15 d	IL-1β<br>(pg/mL)<br>at 7 d	IL-1β<br>(pg/mL) <br>at 15 d	IL-8<br>(pg/mL)<br>at 7 d	IL-8<br>(pg/mL)<br>at 15 d	Albumin<br>(g/dL)<br>pre-op	Albumin<br>(g/dL)<br>at 7 d	Albumin<br>(g/dL)<br>at 15 d
CG (n=65)	32.5	24.3	18.7	12.5	45.2	33.4	3.6	3.4	3.5
OG (n=65)	30.1	19.8	17.2	9.3	43.8	28.9	3.7	3.8	4.1
t-value	2.14	3.25	1.98	3.42	1.78	3.05	1.45	2.89	3.75
P-value	<0.05	<0.01	<0.05	<0.01	<0.05	<0.01	0.15 (NS)	<0.01	<0.001

### Nutritional indicators for patients with improved early enteral nutrition

**Figure 3:**
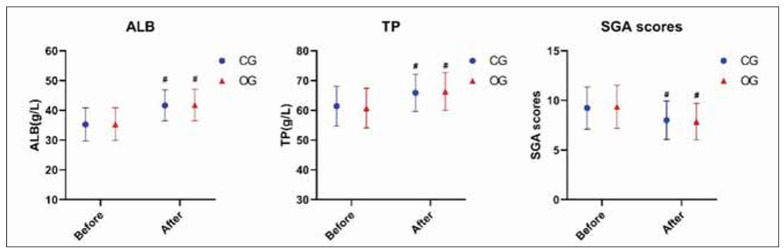
Evaluation of nutritional indicators. ^#^P<0.05 represents before versus after.

As shown in Figure 3, ALB, TP levels and SGA scores were higher after early enteral nutrition support than before treatment (P<0.05). However, the two groups had no significant difference (P>0.05).

### Cranial reconstructive therapy combined with lumbar big-pool drainage reduces patient complications

As shown in Table 3, the number of recurrent cerebrospinal fluid nasolacrimal leaks, fever, intracranial infections, and intracranial hypertension were smaller in OG than in CG, with the greatest variation in recurrent nasolacrimal leaks, and the overall complication rate was significantly lower in OG than in CG (P<0.05).

### Cranial reconstructive therapy combined with lumbar big-pool drainage improves patients’ negative emotions

**Figure 4:**
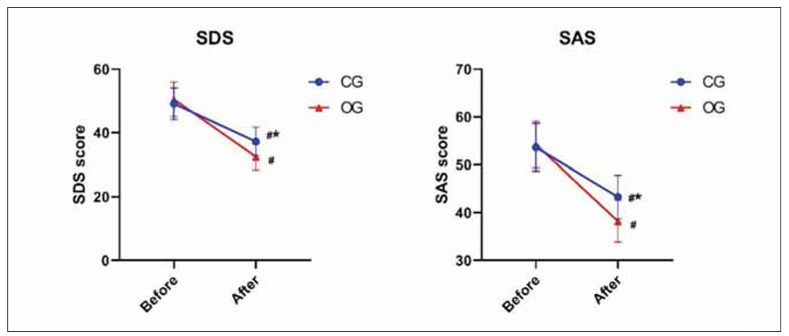
Evaluation of SDS and SAS. ^#^P<0.05 represents before versus after. *P<0.05 represents CG versus OG.

As shown in Figure 4, the SDS and SAS scores were better after treatment than before, and the improvement in OG was better (P<0.05).

### Cranial reconstructive therapy combined with lumbar big-pool drainage improves patients’ QOL

**Figure 5:**
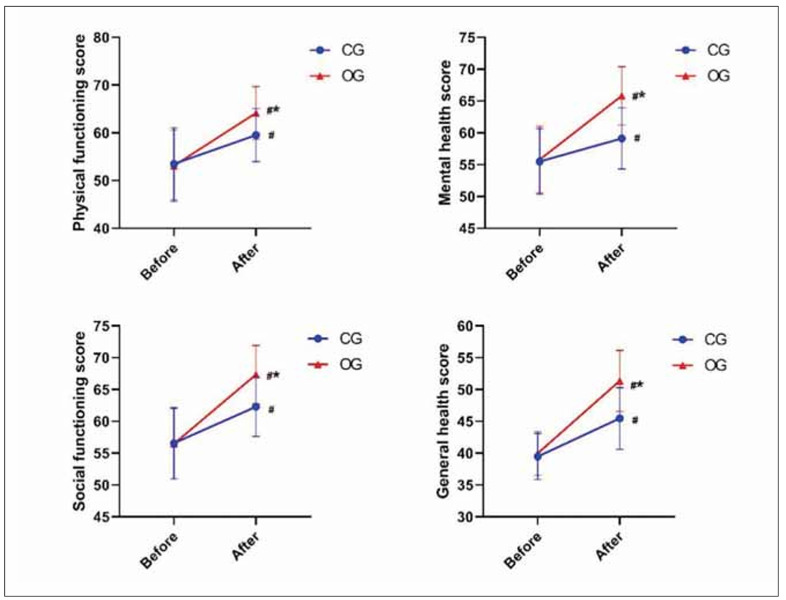
Evaluation of QOL. ^#^P<0.05 represents before versus after. *P<0.05 represents CG versus OG.

As shown in Figure 5, the QOL of both groups was notably better after treatment than before treatment, and the QOL of OG was superior (P<0.05).

## Discussion

Cerebrospinal fluid leakage is a relatively rare lateral skull base disorder seen in both spontaneous and exogenous onset. Exogenous temporal bone injuries are most often seen in traumatic causes [19] [20]. Treatment mainly includes conservative treatment and surgery. Conservative treatment means that the patient stays in bed, head elevated by 30 degrees, is given antibiotics, keeps the intestines open, avoids sneezing and so on. If the bleeding persists for a fortnight after conservative treatment, repair surgery is considered [21] [22].

Our study included patients who underwent repair surgery for temporal bone fractures, and both groups were given nutritional and psychological care. One group was given a craniotomy for repair, and the other group had a lumbar drain placed after surgery. There was no significant difference in the general data of the two groups. Clinical outcomes showed a higher cure rate in CG than in OG, and one case of OG died due to intracranial infection. After the leakage of cerebrospinal fluid, pathogens can easily enter the intracranial channels from the bloodstream, thus causing intracranial infections, and the infection rate is as high as 9% [23] [24]. Therefore, timely fistula closure is the key to reducing intracranial infection. Recurrent postoperative cerebrospinal fluid leakage is not uncommon, and studies have shown that patients’ inattention to bony reconstructive therapy, such as increased intracranial pressure caused by forceful coughing, can cause recurrence [25].

Lumbar large pool drainage, a circulatory pathway established through subarachnoid puncture placement, stabilises the patient’s intracranial pressure and thus promotes accelerated fistula healing and is a commonly used clinical treatment for cerebrospinal fluid leakage. To detect whether cerebrospinal fluid leakage causes some neurological diseases, such as meningitis, we tested the cerebrospinal fluid for protein, glucose and chloride. An increase in cerebrospinal fluid protein suggests bacterial inflammation, an increase in glucose suggests viral ence phalitis, etc., a decrease suggests bacterial meningitis and a decrease in chloride suggests meningitis [26]. In our study, glucose, chloride, and protein were mostly within the normal range in both groups on the seventh postoperative day. The biochemical levels in OG suggested that patients with OG were safer. Antibiotic use has been demonstrated to prevent cerebrospinal fluid leakage infections, but it is controversial in terms of whether it reduces CNS infections [27]. In our study, perioperative antibiotic therapy was administered to all patients. Specifically, the patients received ceftriaxone (2 g IV) and metronidazole (500 mg IV) for 7 days. The results showed that seven days postoperatively, inflammation level indicators such as white blood cell (WBC) count and C-reactive protein (CRP) levels were significantly lower in the observation group (OG) compared to the control group (CG). However, no significant difference in these verified levels was observed after 15 days, suggesting that the perioperative antibiotic regimen effectively controlled infection during the early postoperative period and helped reduce the inflammation associated with the surgical intervention.

In addition, the postoperative lumbar large pool drainage can control the infection time faster and promote the recovery of the patient’s condition.

Patients with temporal bone fracture with cerebrospinal fluid leakage usually have more severe trauma, and some of them are undernourished due to their inability to eat actively. Under stress, the intestinal mucosal barrier function is impaired, and bacteria and toxins can enter the blood circulatory system through the mucosal barrier and pass the blood-brain barrier, leading to intracranial infections. Early enteral nutrition can protect the gastrointestinal tract, balance the intestinal flora, and reduce the chance of bacteria entering the body through the intestinal mucosa, thus improving the clinical outcome [28] [29]. In addition, high-calorie and easy-to-digest foods can promote defecation and reduce the likelihood of constipation or lack of defecation, thus lowering intracranial pressure. Psychological care is also important, as patients are prone to anxiety and other negative emotions due to the long duration of the disease and the tendency to relapse; psychological care can reduce anxiety and depression and significantly improve the patient’s QOL. Our results showed that SDS, SAS and QOL were associated with the clinical outcome and prognosis of the patients and that cranial reconstructive therapy combined with lumbar pool drainage was better than reconstructive therapy alone in all the indicators.

Our findings highlight the significant role of inflammatory cytokines (IL-6, IL-1β, and IL-8) and serum albumin (ALB) levels in monitoring postoperative recovery in patients with temporal bone fractures and cerebrospinal fluid (CSF) leakage. The greater reduction in IL-6, IL-1β, and IL-8 levels in the observation group (OG) by day 15 suggests that cranial reconstructive therapy combined with lumbar drainage more effectively controls systemic and neuroinflammation, potentially lowering the risk of complications such as infection and prolonged CSF leakage. Higher postoperative albumin levels in OG indicate better nutritional recovery, which is critical for immune function, tissue healing, and overall prognosis. Since albumin is a nutritional and inflammatory marker, its improvement in OG further supports the hypothesis that reduced systemic inflammation leads to enhanced metabolic recovery.

The reductions in IL-6, IL-1β, and IL-8 levels observed in the OG are statistically significant and clinically meaningful. Lower levels of these inflammatory cytokines are strongly associated with improved recovery outcomes, as they reflect a decrease in systemic inflammation, which can lead to faster healing and reduced risk of complications. In particular, reduced cytokine levels are linked to a lower likelihood of infection, a faster resolution of CSF leakage, and a reduced risk of prolonged hospital stays or adverse neurological outcomes. The clinical significance of these findings emphasises the potential benefit of combining cranial reconstruction with lumbar drainage to optimise both systemic inflammation control and the overall recovery process in patients with temporal bone fractures and CSF leakage.

These findings emphasise the importance of integrating inflammatory and nutritional monitoring in the postoperative management of skull base fractures, as better control of these parameters may lead to improved patient outcomes and faster recovery. The study by Zeiler et al. [30] focuses on the evaluation of cytokine levels in cerebrospinal fluid (CSF) and cerebral microdialysis (CMD) in severe traumatic brain injury (sTBI) patients, highlighting the role of inflammatory markers in long-term neurological outcomes. This systematic review identifies IL-1β, IL-1ra, IL-6, IL-8, IL-10, and TNF-α as key cytokines associated with patient prognosis at 6–12 months post-injury [30]. Compared to our study, which examined IL-6, IL-1β, and IL-8 in temporal bone fractures with cerebrospinal fluid leakage, the Zeiler et al. [30] review expands its focus to severe TBI, assessing long-term implications rather than short-term postoperative recovery. While both studies recognise the importance of IL-6, IL-1β, and IL-8 in neuroinflammation, our research specifically evaluates their role in postoperative inflammation resolution, showing that lumbar drainage combined with cranial reconstructive therapy accelerates inflammatory marker reduction. Additionally, our study uniquely integrates serum albumin (ALB) as a marker of nutritional status and systemic inflammation, a factor not explored in Zeiler et al.’s [30] review. Despite these differences, both studies emphasise the need for further research on cytokine monitoring in neurological trauma and postoperative inflammation, reinforcing the potential of targeted inflammatory modulation to improve patient outcomes.

In a previous study by Manganotti et al. [31], elevated levels of interleukins IL-6 and IL-8 were observed in both cerebrospinal fluid and serum during the acute phase of COVID-19 neuropathy in patients with Guillain-Barré syndrome [31]. This study highlighted the role of inflammation in neurological complications, where both IL-6 and IL-8 were found to be increased during the acute phase, and IL-8 remained elevated in serum even after recovery. Similarly, our study found that inflammation markers such as IL-6, IL-1β, and IL-8 were significantly reduced in the observation group after cranial reconstruction combined with lumbar drainage. These findings align with the idea that cytokines like IL-6 and IL-8 play an essential role in mediating inflammatory responses and suggest that managing inflammation can improve recovery outcomes in patients with temporal bone fractures and cerebrospinal fluid leakage.

In a previous study by Bodnar et al. [32], the role of inflammation in disrupting the central nervous system (CNS) barriers following traumatic brain injury (TBI) was explored, with a particular focus on the pro-inflammatory cytokine IL-1. The study highlighted that TBI leads to primary physical damage to CNS barrier-forming cells and secondary inflammatory response, further disrupting the blood-brain barrier and other critical barriers. This inflammatory response, driven by cytokines like IL-1, is crucial for remodelling the barriers but can also lead to chronic dysfunction. Similarly, our study showed significant reductions in inflammation markers such as IL-6, IL-1β, and IL-8, suggesting that managing inflammation may help mitigate similar disruptions in the cerebrospinal fluid and related barriers in patients with temporal bone fractures and CSF leakage. Both studies underscore the importance of targeting inflammation as a therapeutic strategy to protect CNS integrity and improve recovery outcomes.

The manuscript acknowledges several limitations, including the short follow-up period and the lack of assessment of other cytokines, such as IL-10 and TNF-α. Unmeasured confounding factors, such as surgical technique variations or patients’ postoperative care, could also influence the study’s results. These factors could potentially affect the outcomes, and their impact should be considered when interpreting the results. Future studies with larger sample sizes, more extended follow-up periods, and more controlled variables could help address these potential confounders.

## Conclusion

Cranial reconstruction combined with lumbar drainage accelerates the resolution of inflammation (IL-6, IL-1β, IL-8), improves cerebrospinal fluid markers, and enhances nutritional recovery, leading to better clinical outcomes. These results suggest that combining lumbar drainage with cranial reconstruction should be considered a standard treatment for patients with temporal bone fractures and cerebrospinal fluid leakage. This approach may reduce complications such as infection and prolonged recovery by improving inflammation control and CSF dynamics. Additionally, integrating nutritional and psychological support can optimise patient recovery and quality of life.

## Dodatak

### Assurance of the originality of data

The author(s) assure the readers and the publishers that all the data presented here are original.

### Conflict of interest statement

All the authors declare that they have no conflict of interest in this work.
